# Allium ureteral stent for the treatment of malignant ureteral obstruction: A median term study

**DOI:** 10.1097/MD.0000000000034309

**Published:** 2023-07-28

**Authors:** Haopu Hu, Mingrui Wang, Xinwei Tang, Chin-Hui Lai, Qi Wang, Kexin Xu, Tao Xu, Hao Hu

**Affiliations:** a Department of Urology, Peking University People’s Hospital, Beijing, China.

**Keywords:** allium ureteral stent, life quality, maintenance therapy, malignant ureteral obstruction, self-expandable metallic stents

## Abstract

This study aimed to assess the safety and efficacy of Allium ureteral stents for the maintenance therapy of malignant ureteral obstruction (MUO). Clinical data of 25 patients (27 sides) with ureteral obstruction caused by a malignant tumor from December 2018 to December 2021 were retrospectively analyzed. Preoperative ultrasonography and computed tomography urography indicated hydronephrosis and MUO. Allium ureteral stents were placed using a retrograde or antegrade approach. Therapeutic effects and complications were recorded. The Wilcoxon signed-rank test was used to compare continuous variables between the preoperative and the last follow-up. A total of 25 patients (27 sides) were included in this study. After a follow-up time of 18 (11–29) months, the width of hydronephrosis [1.6 (1.0–2.2) cm vs 2.6 (1.2–3.3) cm, *P* = .000], glomerular filtration rate [83.8 (58.1–86.4) mL/minutes/1.73 m2 vs 74.5 (56.8–79.1) mL/minutes/1.73 m2, *P* = .001] and score of ureteral stent symptoms questionnaire [77 (76–79) vs 100 (98–103), *P* = .000] was significantly improved. Stent migration occurred in 3 of the 25 patients within 3 months after surgery. All patients with complications were followed up for at least 6 months after stent adjustment or exchange, and no other complications were found. Two patients died because of malignant complications. The stent patency rate was 88.9% (24/27) after the first operation, and 100% (27/27) after complications were treated. The Allium ureteral stent is safe and effective for the maintenance therapy of MUO, which can dramatically relieve the symptoms of patients. Stent migration is a major complication that can be resolved by endoscopic adjustment.

## 1. Introduction

Malignant ureteral obstruction (MUO) secondary to a local primary tumor, metastatic tumor, or retroperitoneal lymphadenopathy, is often identified as one of the classic signs of advanced tumors. MUO can lead to renal failure and prevent some patients from using systemic therapies, which usually results in a shortened life expectancy and prominently reduced quality of life. The median survival time of patients was no more than 5 months, despite proper treatment.^[1]^ Given the frailty and poor prognosis of this patient population, radical treatment may not be of interest. Thus, there is evidence that palliative urinary diversion is effective in preventing renal failure and may provide survival benefits,^[2]^ and can be performed by double-J stent insertion or percutaneous nephrostomy.^[3]^ However, these 2 methods require frequent replacement, resulting in a reduced quality of life. Moreover, percutaneous nephrostomy is associated with high complication rates.^[2]^

**Figure 1.**
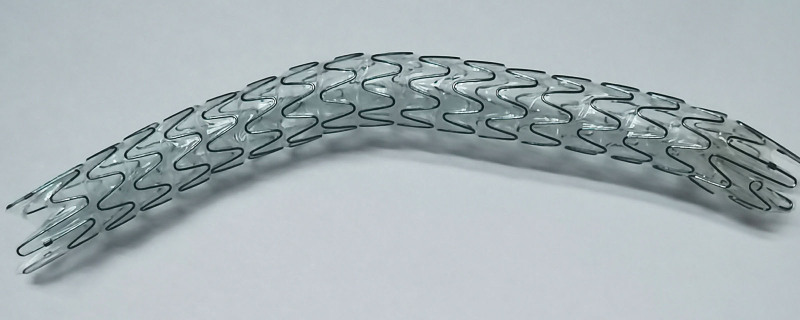
The Allium ureteral stent.

Allium ureteral stent is a type of segmental metal-coated stent that has a large diameter and solid support, can resist the progressive compression of the tumor, and can achieve satisfactory drainage (Fig. 1). The effectiveness of Allium ureteral stents has been proven in retrospective studies. However, they failed to assess changes in the quality of life. Therefore, we retrospectively analyzed the clinical data and follow-up results of patients with MUO treated with Allium ureteral stents in our center and explored the therapeutic potential of this maintenance method in patients with MUO. Moreover, quality of life analysis of patients with MUO was performed using the ureteral stent symptoms questionnaire (USSQ) for the first time.

## 2. Methods

### 2.1. Study population and data

A total of 25 patients, including 6 males and 19 females, who received an Allium stent in the Department of Urology, Peking University People’s Hospital from December 2018 to December 2021, were all consecutive patients. The inclusion criterion was ureteral obstruction secondary to a malignant tumor. The diagnosis of stricture was ascertained by medical history and preoperative radiographic imaging (ultrasonography and computed tomography urography). Exclusion criteria included patients with severe ureteral obstruction, difficulty in inserting a guide-wire retrogradely or anterogradely, uncontrolled acute and chronic inflammation of the genitourinary system, severe hematuria, difficulty in observing the visual field under endoscopy, and poor physical condition that could not tolerate anesthesia or surgery.

### 2.2. Surgical technique

**Figure 2.**
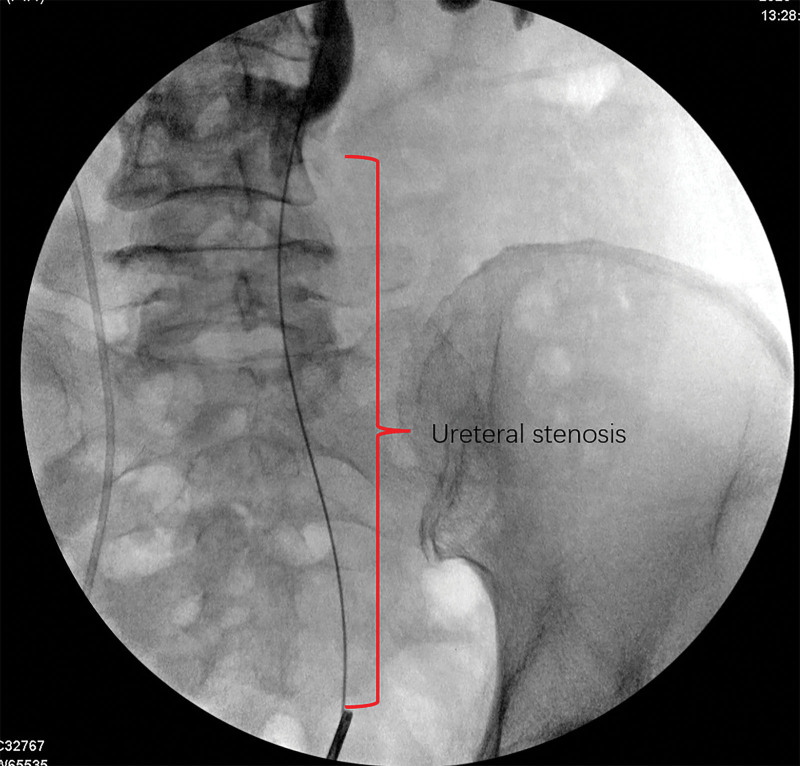
The position and length of ureteral stenosis are determined by retrograde or antegrade ureterogram.

**Figure 3.**
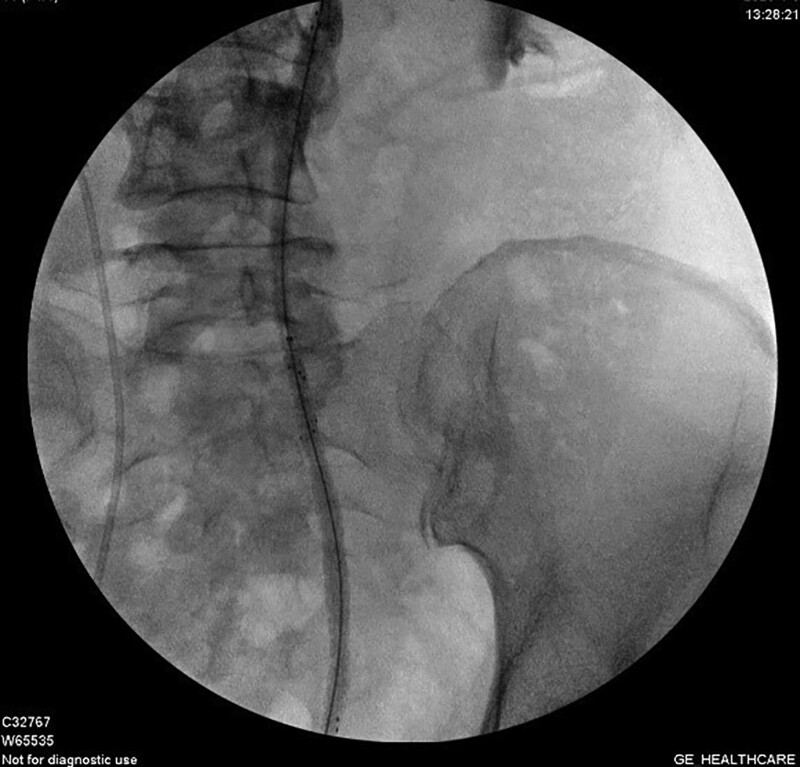
For patients with long stenosis, Allium ureteral stents were inserted in tandem.

All surgeries were performed by the same urologist. The procedure was first conducted in the lithotomy position, under lumbar or general anesthesia. After rigid cystoscopy, a guide-wire was placed into the target ureteral orifice (in the case of a D-J stent being present, the D-J stent should be extracted before inserting the guide-wire.). The location and length of the ureteral stenosis were determined using ureterography (Fig. 2). If the patient had previously treated with percutaneous nephrostomy, contrast media was introduced through the nephrostomy tube. Subsequently, a balloon dilator (BARD, USA, 6cm-21F) was inserted into the obstructed side, and the stenosis segment was dilated to 25 atm for 3 minutes. An Allium stent (12 cm, 24F/30F) was inserted and identified using fluoroscopic imaging. If the ureteral stenosis was too long to be covered by 1 stent, we inserted 2 or 3 stents in tandem, with an overlap longer than 2 cm, to avoid stent migration (Fig. 3).

For patients whose guide-wire failed to pass through the ureteral stricture retrogradely, the operation was changed to anterogradely in an oblique supine position. Under color Doppler ultrasound guidance, we punctured the target calyces and established a 16F channel using a fascia dilator. Then, a guide-wire could pass through the stricture anterogradely. On the other side, the guide-wire was pulled out of the urethra with forceps, followed by F21 balloon dilation and Allium stent insertion, which was the same as the retrograde procedure. As the focus of our study was only on the maintenance therapy for individuals with MUO, we refrained from actively replacing the stents within a period of 3 years after implantation, unless critical stent-related complications or an escalation in ureteral obstruction was detected during post-treatment follow-ups.

### 2.3. Follow-up protocol

On the first postoperative day, KUB (Kidney-ureter-bladder) was performed to confirm the location of the Allium stent. All patients were follow-up at 1, 3, 6 months after surgery and every 6 months thereafter, and were reviewed in advance if obstruction symptoms, such as recurrent fever or persistent lower back pain, occurred. The follow-up indicators included urine tests, urine cultures, and abdominal plain films. Due to the poor physical condition of patients with MUO, renal function was evaluated by hydronephrosis width (the horizontal diameter of the renal pelvis measured by ultrasonography) and glomerular filtration rate (GFR). The USSQ was completed prior to surgery and at each follow-up visit. If hydronephrosis is aggravated, computed tomography urography is needed to exclude complications. The primary outcome indicator was the patency rate of the Allium ureteral stent, as reflected by the difference in GFR levels and size of hydronephrosis at the end of the follow-up period. The secondary outcome indicators were stent-related 90-day postoperative complications and patients’ subjective quality of life, as reported by the USSQ at the end of the follow-up.

### 2.4. Statistical analysis

SPSS v. 27 software (SPSS Inc. IBM Corp, Somers, NY) was used for data analysis. Continuous variables were expressed as medians and IQRs, and categorical variables were described as frequencies (proportions). The Wilcoxon signed-rank test was used to compare continuous variables between preoperative and the last follow-up. Statistical significance was set at *P* < .05.

## 3. Results

In this study, Allium stents were successfully placed in all the 25 patients with MUO (27 sides). The general patient characteristics are shown in Table 1. Of these patients, 6 were male and 19 were female, with a median age of 56 (43–66.5). The most common location of the obstruction was the middle and distal ureters (77.8%), and the median stricture length was 6.5 (3.0–15.0) cm. The most common primary disease that caused MUO was colon cancer in 13 patients (52.0%), followed by cervical cancer in 4 patients (16.0%), bladder cancer in 2 patients (8.0%), ureteral cancer in 2 patients (8.0%), ovarian cancer in 1 patient (4.0%), breast cancer in 1 patient (4.0%), and duodenal cancer in 1 patient (4.0%). Before Allium stent insertion, most patients were previously treated with double-J stents (92.0%), and a minority were treated with percutaneous nephrostomy (8.0%).

**Table 1:** General characteristics of the patients*.

Variable		Overall
No. patients (n)		25
	Male	6 (24.0)
	Female	19 (76.0)
Age (yr)		56 (43–66.5)
BMI (kg/m2)		23.1 (22.4–25.9)
Side (n)		
	Left	14 (56.0)
	Right	9 (36.0)
	Bilateral	2 (8.0)
Stenosis length (cm)		6.5 (3.0–15.0)
Obstruction location (n)		
	Proximal	1 (3.7)
	Middle	6 (22.2)
	Distal	8 (29.6)
	Middle and distal	7 (25.9)
	Whole	5 (18.5)
Primary disease (n)		
	Bladder cancer	2 (8.0)
	Cervical cancer	4 (16.0)
	Colon cancer	13 (52.0)
	Ovarian cancer	1 (4.0)
	Breast cancer	1 (4.0)
	Duodenum cancer	1 (4.0)
	Ureteral cancer	2 (8.0)
	Endometrial cancer	1 (4.0)
Prior treatment (n)		
	Double-J stent	23 (92.0)
	Percutaneous Nephrostomy	2 (8.0)

BMI = body mass index.

* Continuous variables are reported as median (IQR) and categorical variables as number (%).

The features associated with this procedure are shown in Table 2. Allium stents were inserted in all patients with MUO successfully, 8 patients inserted 2 stents and 3 patients inserted 3 stents in tandem due to long ureteral obstructions. The median operative time was 24 (12–48) minutes, and the median postoperative hospital stay was 3 (1–4) days. Perioperative symptoms included pain in 9 patients (36.0%), fever in 3 patients (12.0%) and urinary tract infection in 2 patients (8.0%). The total cost of per patient in our study was $10070.9 ($9862.5–$18876.6) US dollars. The patient with the highest cost of surgery had 3 stents inserted due to severe whole ureteral stenosis, and the final cost was $28,572 US dollars.

**Table 2:** Procedure-related characteristics and follow-up*.

Variable		Overall
Hospital stay time after surgery (d)		3 (1–4)
Total cost ($)		10070.9 (9862.5–18876.6)
Stent number (n)		
	1	16 (59.3)
	2	8 (29.6)
	3	3 (11.1)
Follow-up time (mo)		18 (11–29)
Patency rate (%)		24/27 (88.9)
Patency rate after stent adjusted or exchanged (%)		27/27 (100)
Perioperative complications (n)		
Clavien Dindo grade I		
	Fever	3 (12.0)
	Pain	9 (36.0)
Clavien Dindo grade II		
	Urinary tract infection needed antibiotics	2 (8.0)
Clavien Dindo grade III		
	Stent migration	3 (11.1)

* Continuous variables are reported as median (IQR) and categorical variables as number (%).

Table 2 presents the surgical treatment outcomes. After stent insertion, 24/27 (88.9%) sides remained unblocked owing to improved GFR and decreased renal pelvis width. Stent migration occurred in 3 (11.1%) patients within 3 months after surgery. Two patients with uncomplicated migration underwent endoscopic stent repositioning, while 1 patient presented with restenosis of the ureteric segment at the distal end of the stent, in addition to stent migration. As a result, dilation of the stenotic segment was performed along with stent replacement. All patients who developed complications were followed up for > 6 months after stent adjustment or replacement, and further complications were not observed.

After a follow-up period of 18 (11–29) months, 27/27 (100.0%) stents remained unblocked, including those cases with complications. Two patients died because of malignant complications. A comparison of the main therapeutic indicators is presented in Table 3. The width of hydronephrosis [1.6 (1.0–2.2) cm vs 2.6 (1.2–3.3) cm, *P* = .000] and GFR [83.8 (58.1–86.4) mL/minutes/1.73 m2 vs 74.5 (56.8–79.1) mL/minutes/1.73 m2, *P* = .001] improved obviously after surgery. Meanwhile, the USSQ score [77 (76–79) vs 100 (98–103), *P* = .000] was significantly lower than that before pre-operation with double-J stents or nephrostomy tubes.

**Table 3:** Comparison of main therapeutic indicators*.

Variable	Pre-operation	Last follow-up	*P* value
Hydronephrosis width (cm)	2.6 (1.2–3.3)	1.6 (1.0–2.2)	.000
GFR (mL/min/1.73 m2)	74.5 (56.8–79.1)	83.8 (58.1–86.4)	.001
USSQ score (point)	100 (98–103)	77 (76–79)	.000
Urinary symptoms	34 (34–39)	28 (26–30)	.000
Body pain	19 (15–19)	14 (12–15)	.001
General health	19 (17–21)	13 (13–14)	.000
Work performance	11 (10–12)	10 (8–12)	.029
Sexual matters	2 (2–2)	2 (2–2)	.180
Additional problems	13 (12–13)	10 (9–11)	.000

GFR = glomerular filtration rate, USSQ = ureteral stent symptoms questionnaire.

* Continuous variables are reported as median (IQR).

## 4. Discussion

There was prominent heterogeneity in the etiology and symptoms of patients with MUO. Obstruction may be caused by internal or external compression due to the primary tumor, metastasis, or enlarged lymph nodes. Primary tumors can arise from the urinary system, such as ureter, bladder, or prostate cancer, or from non-urinary systems, such as colorectal, lymphoma, uterine, cervical, ovarian, and breast cancer.^[4]^ If left untreated, MUO can lead to renal failure and may affect systemic treatment of malignancies.^[5]^ In view of the poor prognosis of patients with MUO, palliative or maintenance urine drainage is often adopted, mainly including percutaneous nephrostomy and various types of ureteral stent insertion.^[6]^ MUO is characterized by severe ureteral stricture, long stenosis length, and progression to primary tumors, contributing to the failure of traditional treatment.

Of the various maintenance treatment options available, percutaneous nephrostomy offers a relatively high success rate (98.7% vs 89%) compared to retrograde stenting. Moreover, it can be performed under local anesthesia, making it a viable alternative for patients who cannot tolerate general or partial anesthesia. However, owing to its considerable impact on quality of life and the requirement for an external appliance, percutaneous nephrostomy is usually reserved as a second-line therapy in cases where retrograde stent placement is not feasible.^[7–9]^ Meanwhile, percutaneous nephrostomy treatment has a 10% chance of resulting in severe complications such as sepsis and bleeding, and is burdened by the disadvantage of requiring tube replacement every 2 to 3 months.^[10]^

The polymeric double-J stent is an appealing first choice for the management of ureteral strictures. This method is readily available and widely used by urologists without requiring specialized facilities or equipment. However, polymeric stents may fail in approximately 20% of cases and can lead to complications such as urinary tract infections, encrustation, and obstruction.^[11,12]^ Therefore, the stent must be replaced every 3 months.^[11]^ Tandem stenting enhances the efficacy and durability of conventional double-pigtail stents in patients with MUO. This approach has already shown significant patency rates in patients with limited survival estimated between 3 and 5 months; however, an increase in urinary tract symptoms is expected, highlighting the need for further exploration of more appropriate stent design structures.^[13,14]^

Metal ureteric stents are designed to be more effective in maintaining lumen patency and can be used as salvage treatment after the failure of double-J stents. Reducing the frequency of exchange and stent-related symptoms offers a new option for patients with MUO, especially those with fragile physical conditions that are not suitable for frequent anesthesia or patients undergoing oncotherapy.^[15]^

The Allium stent (Allium, Israel) is a large-caliber self-expanding stent composed of Nitinol alloy that is externally coated with a thin layer of copolymer to prevent intraluminal ingrowth and allow for a prolonged indwelling time and easy removal. Studies conducted by Moskovitz et al^[16]^ have shown that the use of Allium stents in patients with stenosis caused by gynecological or bladder malignant tumors leads to a low restenosis rate (0%), with only 10% of the stents experiencing migration 3 months after insertion. Additionally, the average indwelling time of Allium stents was 17 months, which is longer than that of other metal stents. A self-expanding metal ureteral stent may be a more effective treatment for the management of patients with MUO with long expected survival.

According to our research on 25 patients (27 sides of ureteral obstruction) with an average follow-up period of 18 (11–29) months, the drainage patency rate after the initial Allium stent insertion was 88.9% (24/27), and no serious stent complications were observed. Among the 3 patients who experienced stent migration, fluent drainage was achieved following stent position adjustment. After adjustment, the patency rate of stented drainage was 100% (27/27) during the follow-up period exceeding 6 months, which is consistent with the findings reported by Moskovitz et al.^[16]^ Additionally, the success rate of long-term indwelling of Allium stents in our study was higher than that of other commonly used metal ureteral stents in patients with MUO, including Resonance (79%), Uventa (81%), and Memokath (65%) stents.^[15]^ By contrast, Resonance stents are more appropriate for patients with proximal or middle ureteral obstruction without high intravesical pressure caused by extensive pelvic or intravesical disease. The median stent duration of Resonance stent was 8.2 months.^[15,17,18]^ Uventa stents are less prone to migration (3%), but are associated with serious complications such as ureteral artery fistula, ureteroenteric fistula, and ureterovaginal fistula in women with cervical cancer, long ureteral stenosis (>6 cm), and long placement time (>24 months). The median follow-up time of existing studies on Uventa stents was 20.3 months, with a mean success rate of 74.5%.^[19]^ Memokath stents, with a median stent duration of 10.5 months, have a lower patency rate of long-term drainage in patients with MUO, and are relatively contraindicated for strictures involving the pelvi-ureteric junction or ureteric orifice.^[20]^ Our study demonstrated that Allium stents are suitable for patients with MUO with multiple etiologies and exhibit good efficacy in patients with different stenosis sites, indicating stable drainage ability in patients with varying conditions.

The majority of the patients in our study had previously undergone treatment with double-J stents before the insertion of Allium stents, and we observed an improvement in follow-up indicators in terms of both renal function and quality of life outcomes. Regardless of the cause of ureteral stricture, timely relief of obstruction can help to protect kidney function.^[21]^ Although conventional double-J stents are effective, they have limitations. Chen et al^[22]^ conducted a study comparing the safety and efficacy of conventional and metal stents, and found that metal stents demonstrated a higher patency rate at 6 months (100% vs 83.8%) and 1 year (91.7% vs 40.0%) than conventional stents. Furthermore, metal stents exhibited a lower overall complication rate than conventional stents (36.7% vs 63.6%), along with higher quality of life scores (30.9 ± 2.8 vs 23.6 ± 1.8).^[22]^ Allium stents may be considered a viable salvage treatment option for patients with MUO who have been treated with double-J stents because of their potential benefits over conventional stents.

Notably, the mortality rate in our cohort was significantly lower than the previously reported rates in patients with MUO.^[1]^ This may be due to our particular strategy for patient selection. In practice, we tend to recommend the use of the Allium stent as a drainage option for MUO patients who have relatively stable tumor treatment and fewer underlying diseases. Most MUO patients with a short life expectancy prefer to avoid stent replacement until death. Therefore, the longer the life expectancy of these patients, the higher the cost-effectiveness of retaining the Allium stent.^[15]^ In contrast, Allium stents ensure tumor treatment for patients with MUO and reduce the mortality rate of patients. Certainly, larger and more comprehensive studies on health economics issues need to be conducted in the future.

However, stent migration is the main complication associated with the use of Allium stents. In our study, the stent migration rate was 11.1% (3/27), which falls within the range of previously reported migration rates (between 10.3% and 23.6%) when followed up for a similar duration.^[16,21,23]^ It is interesting to note that the displaced stents in this study were all located at the distal end of the ureter, which may be due to the severe compression of the distal ureteral stenosis. This can cause uneven force on the stent, leading to its migration. Additionally, a large-scale prospective study of Allium stents identified distal ureteral obstruction as an independent risk factor for stent failure.^[21]^ In this study, patients with stent migration were followed up for > 6 months after stent adjustment, and no other complications were found. Stent adjustment can effectively prevent stent migration and maintain drainage.

According to our study, most patients were successfully drained within 12 months or had unobstructed drainage after stent position adjustment, with a median total cost of $10070.9 ($9862.5–$18876.6) US dollars. Three patients incurred an average total cost exceeding $28,000 US dollars, all of whom had severe strictures across the entire ureter, necessitating the insertion of 3 Allium stents during the procedure. He’ctor et al reported that the cost of their Resonance metallic stent for treating ureteral stricture is comparable to ours, with an annual cost of $13,633.^[24]^ Additionally, the authors discovered that despite metal stents being pricier than polymer stents, the former was linked with a 43% decrease in annual cost ($13,633 vs $23,999).^[24]^ This estimate does not consider savings regarding frequent stent replacement, such as time off work due to hospital visits.

This study had some limitations. First, it was a retrospective study, and only 25 cases (27 sides) were included. Although good efficacy and safety were observed, this still needs to be confirmed in a large-scale prospective study. Second, there was no direct comparison between Allium stents and other treatments.

## 5. Conclusion

Allium ureteral stent is a safe and effective method for the maintenance therapy of patients with MUO, which provides a satisfactory patency rate and significantly improves patients quality of life.

## Author contributions

**Conceptualization:** Haopu Hu, Mingrui Wang, Xinwei Tang, Chin-Hui Lai, Qi Wang, Kexin Xu, Tao Xu, Hao Hu.

**Data curation:** Haopu Hu, Mingrui Wang, Xinwei Tang, Chin-Hui Lai, Qi Wang.

**Formal analysis:** Haopu Hu, Qi Wang.

**Investigation:** Haopu Hu, Mingrui Wang, Xinwei Tang, Hao Hu.

**Methodology:** Haopu Hu, Hao Hu.

**Project administration:** Haopu Hu, Chin-Hui Lai.

**Resources:** Haopu Hu, Mingrui Wang, Chin-Hui Lai, Qi Wang, Kexin Xu, Tao Xu, Hao Hu.

**Supervision:** Haopu Hu, Hao Hu.

**Validation:** Haopu Hu, Chin-Hui Lai, Kexin Xu, Tao Xu.

**Visualization:** Haopu Hu.

**Writing – original draft:** Haopu Hu, Mingrui Wang, Xinwei Tang.

**Writing – review & editing:** Haopu Hu, Mingrui Wang, Kexin Xu, Tao Xu, Hao Hu.
